# An ecological approach to honeybee olfactory conditioning: challenges and potential for the monitoring of potato virus Y infection

**DOI:** 10.1242/bio.061680

**Published:** 2025-09-15

**Authors:** Lorenzo Corsico, Thibault P. M. Costaz, Jolanda Tom, Martin Verbeek, Aria Samimi, Klaas van Rozen

**Affiliations:** ^1^ InsectSense, Plus Ultra-II Building, Bronland 10, 6708 WH Wageningen, The Netherlands; ^2^ Wageningen University & Research, Business Unit Field Crops, Edelhertweg 1, 8219 PH Lelystad, The Netherlands; ^3^ Agroscope, Route des Eterpys 18, 1964 Conthey, Switzerland; ^4^ Wageningen University and Research, Business Unit Biointeractions and Plant Health, Droevendaalsesteeg 1, 6708 PB Wageningen, The Netherlands

**Keywords:** *Apis mellifera* (honeybees), Potato virus Y, Detection, Associative learning, Crop health, Pavlovian protocol

## Abstract

Potato (*Solanum tuberosum* L.) is a global crop with a vital role in food security and economic significance in many countries. Potato virus Y (PVY) is one of its major viral threats, causing severe yield and quality losses when not controlled properly. PVY spreads primarily through aphids and infected tubers, and current management relies on insecticides and protective oils. Novel detection methods are needed to identify infected plants accurately at an early stage of plant development, thus reducing pesticide use. Trained honeybees (*Apis mellifera* L.) can detect specific volatiles emitted by plants infected by viruses like PVY. Using associative conditioning and the proboscis extension reflex, we tested the capacity of harnessed worker bees to distinguish PVY-infected and healthy potato leaves as a first step towards field application. As a whole, the results were impeded by low response levels and no significant result was obtained. However, we were able to show the capability of honeybees to learn and differentiate between two conditioned stimuli (healthy potato leaves versus clean air). Our findings therefore suggest that honeybees, as a globally accessible resource, have the potential to be used as a cost-effective solution in crop health monitoring, with further investigation and protocol refinement needed to achieve accurate PVY detection in agricultural settings.

## Teaser

**Summary:** Hymenopteran insects such as honeybees show great potential as biosensors. We assessed this potential in detecting potato virus Y, exploring a cost-effective approach for monitoring plant health.

## INTRODUCTION

Potato (*Solanum tuberosum* L., Solanaceae) is an irreplaceable crop worldwide. In 2020, the crop was cultivated on more than 20 million hectares, with a global production of 359 million tons (https://www.fao.org/newsroom/detail/doubling-global-potato-production-in-10-years-is-possible/en accessed 16 February 2024). Its production is of crucial importance for world food safety and is of great economic importance for many countries.

Potato virus Y (PVY) is one of the most significant viral pathogens, posing a substantial threat to various crops within the Solanaceae family (Karasev and Gray, 2013; Tsedaley, 2015). The disease induced by PVY in potatoes manifests in the foliage, but certain isolates of the virus can also induce necrosis in tubers, impacting production through significant yield reduction and compromising quality, when not controlled properly. It has a profound impact on agricultural productivity with average annual economic losses on potato production estimated at 187 M EUR for the European Union. These losses are attributed mainly to the costs of chemical treatments and yield drops in both seed and ware potato production (Dupuis et al., 2024).

PVY is primarily transmitted by aphids, although vertical transmission through the vegetatively propagated potato tubers also plays a crucial role in its dissemination (Karasev and Gray, 2013). Management strategies of the virus are mainly done via insecticides, or by applying protective mineral oils on the crop to minimize virus transmission by aphid vectors. Additionally, cultural practices, such as rotating planting locations and roguing (the manual removal of PVY-infected plants), further contribute to the reduction of disease spread (Tsedaley, 2015). In many countries, seed potatoes are under strict seed certification programs. Growers tend to select infected plants as soon as possible and remove them from the field. Before harvest, the potato plants are inspected visually by field inspectors. Post harvest, laboratory tests are used to detect PVY in tuber samples to determine the actual percentage of infected tubers in the harvested seed lot. Novel detection techniques may assist in accurately identifying infected plants in the field, in order to improve effective management by roguing PVY-infected plants, thereby reducing outbreaks, economic damage as well as yield losses. Honeybees (*Apis mellifera* L., Hymenoptera: Apidae) possess remarkable olfactory abilities and learning capacities, making them able to detect specific volatile compounds within complex blends even at low concentrations (Laska et al., 1999; Wright and Smith, 2004). The olfactory system of honeybees is finely tuned to detect and discriminate a diverse array of odours, ecologically crucial for tasks such as locating high quality floral resources (Giurfa, 2007; Paoli and Galizia, 2021; Twidle et al., 2015). Associative learning uses the proboscis extension reflex (PER) of hungry bees towards sugary solutions such as nectar and combines it with specific odour cues (Bitterman et al., 1983; Menzel and Bitterman, 1983). The bees associate the odour with the reward during the conditioning phase. After conditioning, the odour alone can trigger the PER, demonstrating the bee's learned association (Giurfa, 2007; Piqueret et al., 2023). Using this methodology, honeybees were able to discriminate the presence of Mediterranean fruit fly (*Ceratitis capitata* Wiedemann, Diptera: Tephritidae) larvae in oranges, as well as human pathogens such as *Mycobacterium tuberculosis* and SARS-CoV-2 (Chamberlain et al., 2012; Kontos et al., 2022; Suckling and Sagar, 2011).

Volatile organic compounds (VOCs) produced by plants play an essential role in signalling messages between plants as well as insects across trophic levels (Dorokhov et al., 2014). Viruses, such as the potato leaf roll virus (PLRV) and the barley yellow dwarf virus (BYDV) induce specific VOC emission in infected plants (Dorokhov et al., 2014; Jiménez-Martínez et al., 2004; Ngumbi et al., 2007). Similarly, PVY-infected potato plants produce a different volatile blend, especially in the content of β-barbatene and benzyl alcohol, than healthy potato plants (Petek et al., 2014). Honeybees' remarkable olfactory sense, coupled with their ability to learn and remember, holds promise for developing innovative approaches to disease detection and management in agriculture. We believe that honeybees could help disease surveillance in potato crops, offering farmers a cost-effective and efficient means of monitoring plant health.

The objective of this project was to investigate the capability of honeybees to discern between PVY-infected and healthy potato plants while implementing an ecological approach to associative learning. We utilized the well-established Pavlovian conditioning protocol to proof-test their ability to learn and discriminate between samples of healthy potato leaflets, PVY-infected potato leaflets and empty samples.

## RESULTS

### Learning curves

All bees were checked for PER response to sucrose before the conditioning and only bees that displayed a PER were included in the sample. Bees that perished during the conditioning phase were removed from the analysis (Table 1).

**Table 1.** Number of bees collected and tested per day and protocol (i.e. collected bees and tested bees, respectively) with the number of individuals that perished during the learning phase (i.e. perished)

Protocol	Date	Collected bees	Perished	Tested bees
**1**	13 June 2023	30	4	77
**1**	14 June 2023	30	1	
**1**	21 June 2023	30	8	
**A**	23 May 2023	20	3	54
**A**	24 May 2023	20	2	
**A**	25 May 2023	20	1	
**B**	15 June 2023	30	4	84
**B**	20 June 2023	30	2	
**B**	22 June 2023	30	0	

**Fig. 1.**
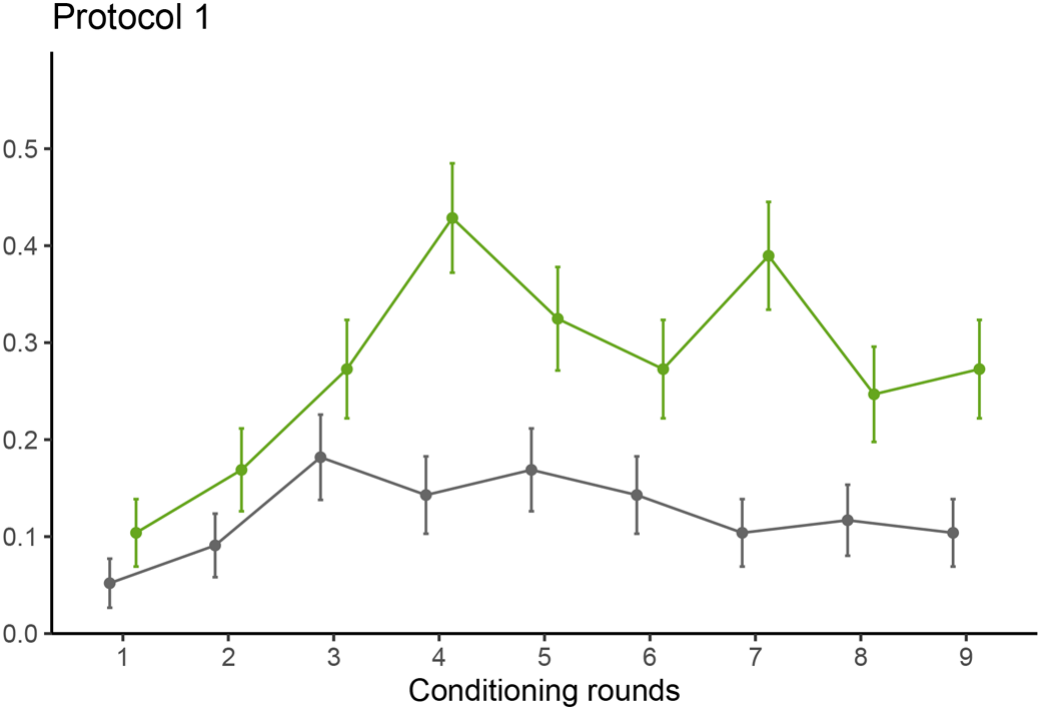
**Bee learning curve (over nine rounds) for protocol 1.** The proportion of PER of the bees is displayed per conditioning round with standard error for proportions as error bars (*n*=77). The green line represents the healthy potato sample (CS^+^), while the grey line represents the empty sample (CS^−^).

**Fig. 2.**
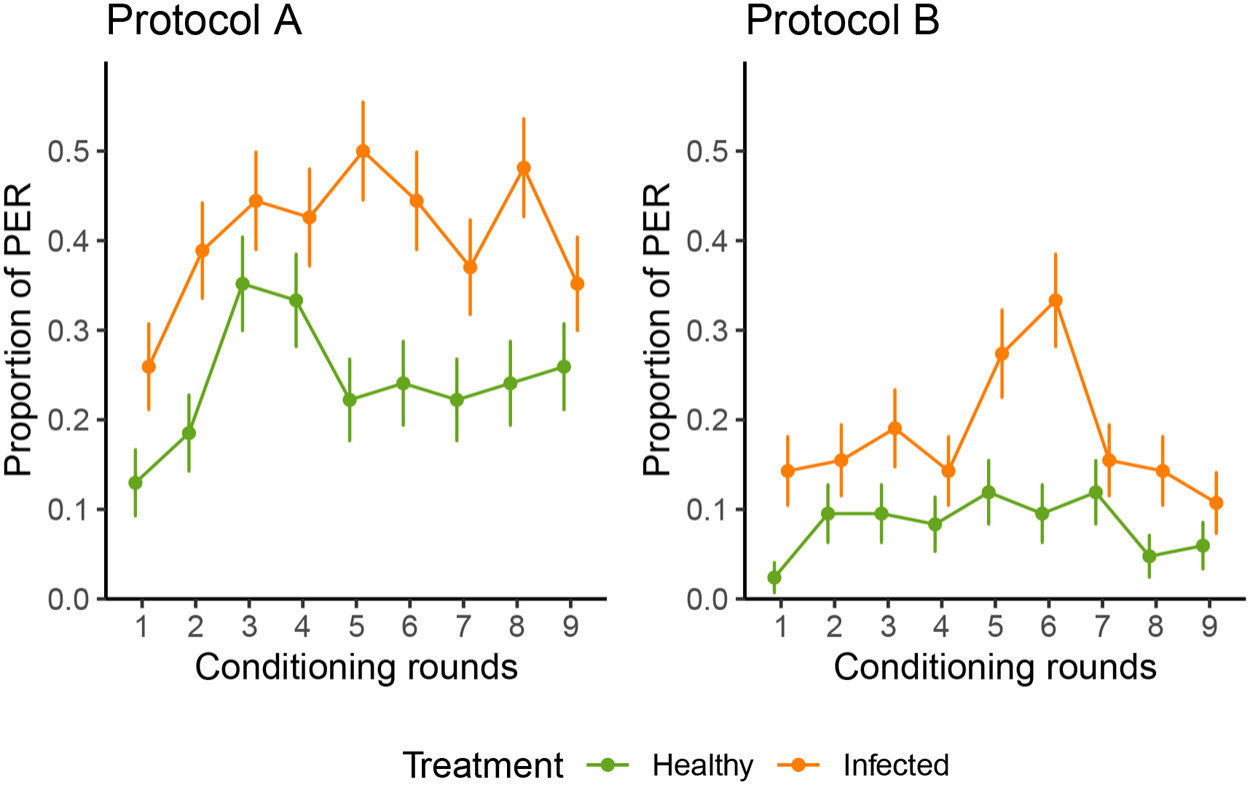
**Bee learning curves (over nine rounds) for protocols A and B.** The proportion of PER of the bees is displayed per conditioning round with standard error for proportions as error bars (*n*=54 and 84 for protocols A and B, respectively). The orange (top) line represents the infected potato sample (CS^+^), while the green (bottom) line represents the healthy potato sample (CS^−^).

Across the 18 trials, the average PER response to the conditioned stimuli (CS) was inferior to 50% in all protocols (Figs 1 and 2), meaning that fewer than half of the tested bees displayed proboscis extension response during the conditioning phase. Thus, the results should be interpreted with caution.

Protocol 1 demonstrated the capability of honeybees to learn the difference between a healthy potato leaf sample and an empty sample (Tukey's post hoc test, *P*<0.001). Average PER was 24.0% (95% confidence interval, CI: 19.3%-29.6%) when exposed to a healthy potato leaf sample with reward against 9.3% (95% CI: 6.8%-12.5%) for an empty sample with no reward. The interaction between conditioning rounds was removed but a positive relationship effect of the conditioning round alone was detected on the PER response (generalized linear mixed model, GLMM, *P*=0.014) (Fig. 1; Table S1).

In protocol A with the overnight starvation (bees were collected at the end of afternoon the day before testing), the bees successfully associated the infected sample (CS^+^) to the reward in contrast with the healthy (CS^−^) sample (Tukey's post hoc test, *P*<0.001). The average PER for the CS^+^ was 33% (95% CI: 21.5%-46.0%). The interaction between the detection of the odour sample and the conditioning round was removed from the model and the round had a standalone positive relationship with PER (GLMM, *P*=0.12) (Fig. 2; Table S2).

Protocol B with a 3 h starvation (bees were collected the same day, during the morning, of the testing) showed similar results as in protocol A, with bees successfully discriminating between the healthy (CS^−^) and infected (CS^+^) samples (Tukey's post hoc test, *P*<0.001) with an average PER of 11.7% (95% CI: 8.1%-16.7%) for the CS^+^ cue with reward. No effect of the conditioning round was detected (GLMM, *P*=1.00) (Fig. 2; Table S3).

It is worth noting that the learning curve (i.e. round effect) marks a plateau before decreasing in later rounds in all the tested protocols. A binomial additive mix-modelling with a smoother on rounds and a random factor for bee ID per day confirmed the non-linearity of rounds for all three protocols with a significant effect of the smooth term on conditioning rounds (e.d.f.=3.52, *P*<0.001 in protocol 1; e.d.f.=3.36, *P*=0.004 in protocol A; and e.d.f.=3.48, *P*<0.001 in protocol B). This could be interpreted as a decrease in appetitive learning towards the sucrose solution or a type of numbness of the harnessed bees after an extended period.

### Memory retention

**Fig. 3.**
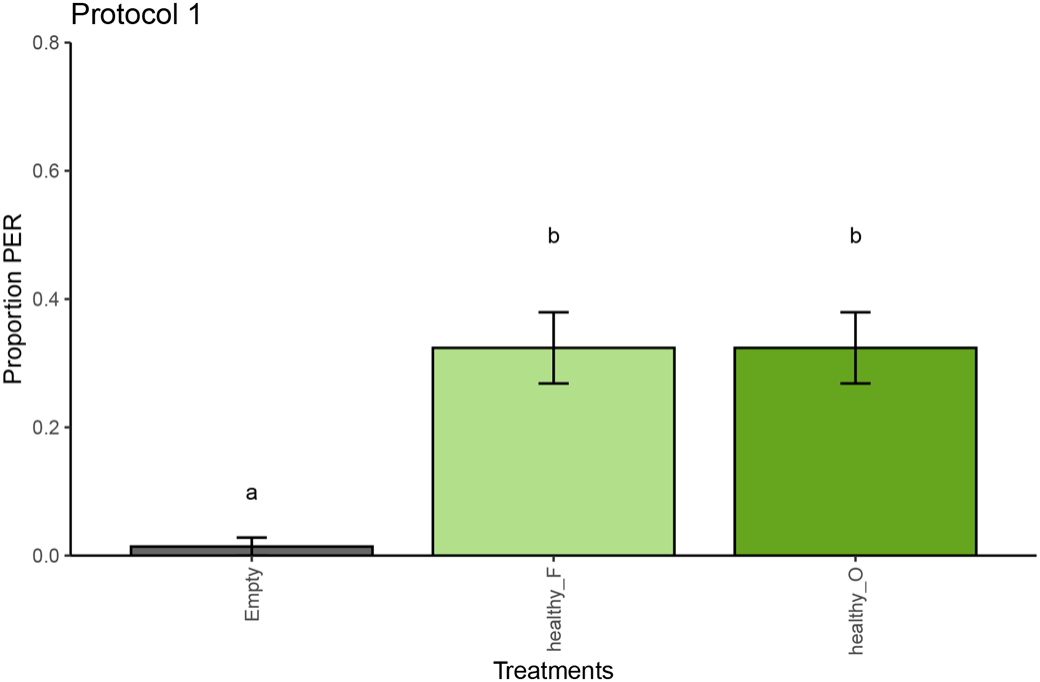
**Memory retention of the conditioned bees for protocol 1 (*n*=71).** The proportion of PER of the bees is displayed per sample type (empty represents ambient air, healthy_O represents old healthy leaf samples, and healthy_F represents fresh healthy potato leaf samples) with standard error around the proportion as error bars. Different letters above the columns represent significant differences between the samples.

**Fig. 4.**
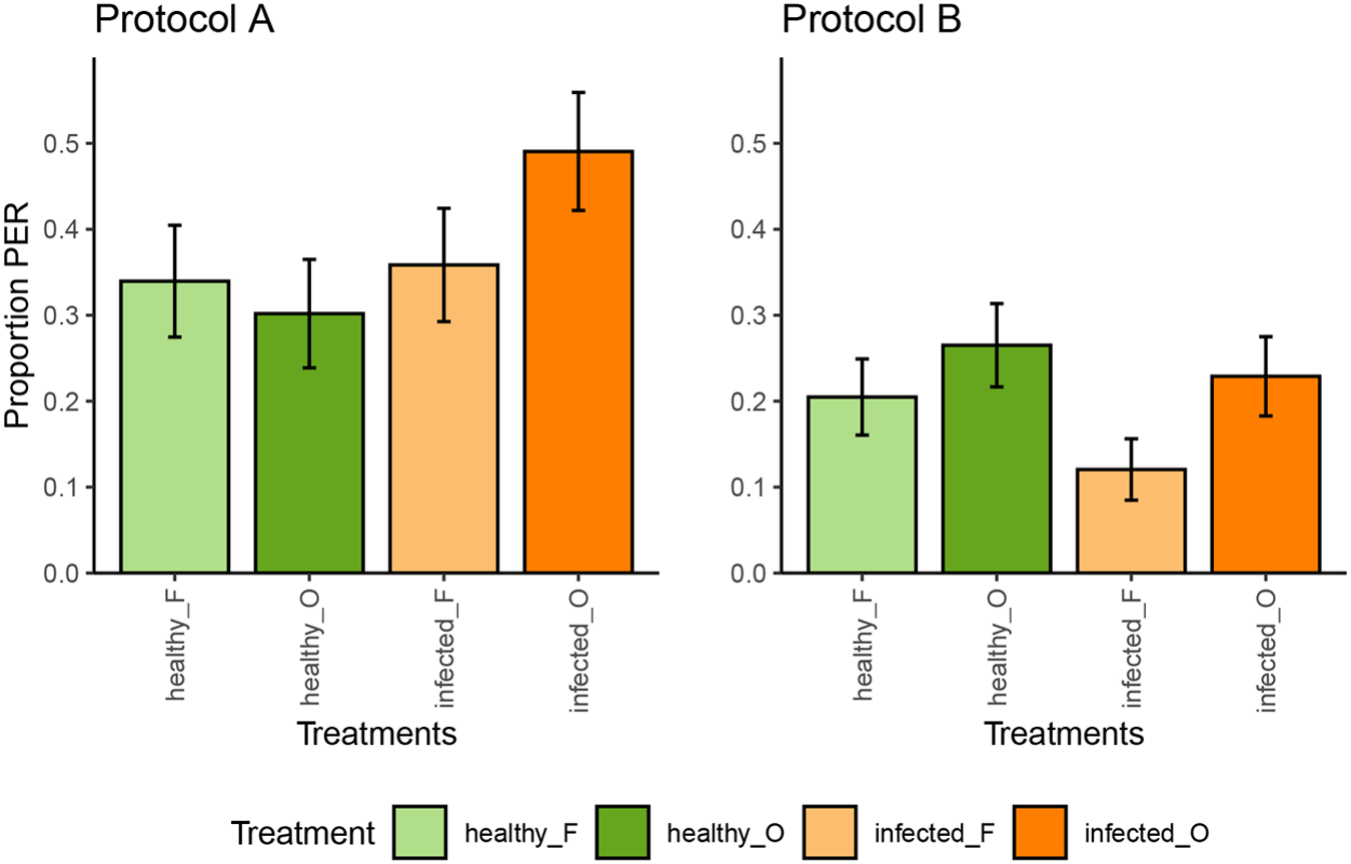
**Memory retention of the conditioned bees for protocols A and B (*n*=53 and 83, respectively).** The proportion of PER of the bees is displayed per sample type (healthy_O represents old healthy leaf samples, healthy_F represents fresh healthy leaf samples, infected_O represents old infected leaf samples, and infected_F represents fresh infected leaf samples) with standard error around the proportion as error bars.

After the training phase, we proceeded with testing the honeybee memory retention (Table 2). In protocol 1, the bees successfully retained the distinction between the healthy potato leaf sample and the empty sample (GLMM, *P*=0.014). Conditioned bees showed 23.4% average PER responses (95% CI: 12.43%-43.0%) towards healthy potato sample for both old and fresh, respectively, against an average of 0.0% (95% CI: 0.0%-0.05%) PER expression on an empty syringe (Fig. 3; Table S4). In protocol A, conditioned bees did not memorize or successfully differentiate the infected potato leaflet odour from the healthy samples (GLMM, *P*=0.13) (Fig. 4; Table S5). Similarly, in protocol B, the memory retention towards the CS^+^ cue was not detected but marginally significant only (GLMM, *P*=0.068) (Fig. 4; Table S6).

**Table 2.** Number of bees tested per day and protocol (i.e. tested bees) with the number of individuals that perished during the memory retention phase (i.e. perished)

Protocol	Date	Bee sample (after conditioning)	Perished	Tested bees
**1**	13 June 2023	26	1	71
**1**	14 June 2023	29	2	
**1**	21 June 2023	22	3	
**A**	23 May 2023	17	1	53
**A**	24 May 2023	18	0	
**A**	25 May 2023	19	0	
**B**	15 June 2023	26	1	83
**B**	20 June 2023	28	0	
**B**	22 June 2023	30	0	

## DISCUSSION

The objective of this study was to investigate whether honeybees could demonstrate positive memory retention towards PVY-infected potato leaflets. However, our findings did not reveal evidence of positive memory retention in response to the conditioned cue. Despite employing various methodologies, we did not observe a significant association between the conditioned cue and positive memory retention with the exception of the healthy versus ambient air cues. Here, we discuss the potential explanations for the lack of positive results in our study. We also propose ameliorations that we believe are better suited for employing honeybees as biosensors in on-field monitoring of crop health.

These results contrast with previous studies that have successfully applied associative learning on harnessed honeybees and conditioned them to detect various olfactory cues (Giurfa and Sandoz, 2012). PER conditioning is a robust methodology; however, most studies took place in a highly controlled environment with a pre-defined concentration of volatiles soaked into a cotton pad (Chamberlain et al., 2012; Kontos et al., 2022; Sandoz et al., 2001). In perspective of field applications, our study adopted an ecological approach to honeybee olfactory conditioning. The infected potato leaf samples were taken from a PVY-infected potato plant. The plants were grown from infected tubers (secondary infection) and all leaves were showing clear PVY-related symptoms. However, concentrations of PVY particles in the selected potato leaves may vary. This choice was made to maintain naturally occurring variations of PVY infection levels found in fields. Moreover, the leaf cuttings may have lost turgescence over time, thus the volatile concentration and blend could have substantially changed between the beginning and the end of the behavioural assay [for a guide on plant volatile extraction see Tholl et al. (2006)]. We recommend using a full potato plant in later assays as the emission of VOCs from the healthy and infected plants are likely to better represent olfactory cues encountered in field situations, thereby enhancing the ecological relevance of the results. We did not use a controlled airflow or air filtering system while these are often present in other studies to ensure that no persistent odours are disturbing the association with the olfactory cue (Chamberlain et al., 2012; Kontos et al., 2022; Suckling and Sagar, 2011). Although these improve the distinction between volatile cues, natural situations are best described by complex blends of VOCs from plants that are most likely responding to a range of abiotic and biotic stresses (Aartsma et al., 2017). It is important to assess the bee performance in targeting the infected plants in field or semi-field situations, as conditions are likely to be highly variable.

Protocol 1 demonstrated the capability of worker honeybees to learn specific odour cues and differentiate between a potato leaf sample and clean air through conditioning. Despite the lack of significant results, protocol A also showed a positive trend in the distinction of a healthy potato leaf sample and an infected potato leaf sample. The capability of worker bees to detect PVY-infected potato plants through associative conditioning aligns with prior research demonstrating the remarkable olfactory sensitivity of worker honeybees (Bitterman et al., 1983; Pham-Delègue et al., 1997; Twidle et al., 2015; Wright and Smith, 2004). As a whole, however, the results of this work need to be taken with caution due to the weakness in the detected trends, which must be ascertained with further testing.

We detected a weakening of the learning curve after the sixth round out of the nine conditioning rounds. However, previous studies showed that using fewer conditioning rounds such as four or five rounds (Sandoz et al., 2001; Suckling and Sagar, 2011), should be sufficient to successfully condition the bees. Additionally, the response level (i.e. the percentage of individuals displaying PER) found in our study was low and hindered the results and their statistical interpretation. The variability in responsiveness is not uncommon in ecological behavioural studies and can be due to a large number of environmental factors such as atmospheric pressure, temperature, and artificial light intensity to cite only a few of them (Alves et al., 2015; Erber et al., 2006). Moreover, the use of one hive only for this study corresponded to a limited genetic diversity in the collected honeybee sample, which might negatively impact the honeybee learning capabilities and the reliability of the results.

Despite these significant limitations, our results suggest that honeybees have the potential to discern between PVY-infected and healthy potato leaf samples in a practical and complex odour habitat, although further investigation is necessary to confirm this trend. Protocols for such investigations need to be tailored to align with the ultimate objective, which is the cost-effective detection of PVY-infected potatoes in field settings. This necessitates the ability to train large numbers of foraging honeybees and identify, on a field scale, the plants to which these conditioned workers are attracted. Moreover, the conditioning methodology should be streamlined and time efficient, moving away from the manual training approach utilized in this study.

The methodology must account for the inevitable ‘mistakes’ made by some individuals, as no olfactory cue study has achieved 100% success. Therefore, a time-efficient method for conditioning large numbers of bees and deploying an on-field tracking system is essential. Moreover, the use of 3D-printed harnesses might represent a constraint for the bees and a potential source of stress that might affect the learning performance. We recommend training entire hives to recognize PVY-infected potato plants using artificial feeders placed on infected potted plants, allowing free-flying bees in semi-open or open designs (Decourtye et al., 2004; Laloi et al., 2000). The conditioned bees would be tagged to facilitate on-field location since they would fly and land near the source of volatiles, creating detectable hotspots on a map (Piqueret et al., 2023). Similarly, trained honeybees could be released in the area of interest and tracked through an unmanned aerial vehicle (UAV) camera or a bee-mapping light detection and ranging (LIDAR) system to create spatial density maps to pinpoint the infected plants within the field (Bromenshenk et al., 2015; Filipi et al., 2022). Furthermore, the social behaviour of honeybees and bumblebees, and their ability to share information within the colony, can further enhance conditioning efficiency (Bridges et al., 2023; Chittka and Rossi, 2022).

We believe that honeybees or other hymenopteran insects could be valuable in the monitoring of crop health and might provide precise detection of specific diseases or pests using plant volatile compounds. Additionally, as a globally accessible resource, honeybees would provide an alternative to the current disease screening methods in the field, which are visual inspection and roguing. Sometimes, on-site tests such as lateral flow devices can be used. These tests are applied on each suspected plant and thus are not cost effective. A large-scale use of honeybees for this application could be relevant for the early detection of infected plants and the removal of these source plants, thereby preventing further spread of PVY by aphids. These plants may be secondarily infected (when the plant is growing from an infected tuber) and act as early virus sources in the field. Moreover, symptoms of PVY are not always shown by the plant due to variety traits, or simply not observed due to weather conditions. Overall, the early detection by bees might decrease the risk of further transmission. In this regard, since climate change is causing invasive pests to reach countries without the facilities to withstand them (Schneider et al., 2022), the availability of cheaper detection methods represents a great opportunity to face such threats. Nonetheless, operational considerations include the fact that bees do not forage at night or in adverse weather conditions, which limits their operational times. Additionally, it remains uncertain whether bees will systematically screen the entire field or focus on the strongest VOC sources. To optimize this methodology, protocols should be adjusted to enhance efficiency, taking into account insect behaviour and environmental constraints. Advanced tracking technologies can improve accuracy and coverage in monitoring bee movements, and training entire hives can enhance collective learning and detection success rates. By addressing these factors, bees could be effectively used for on-field tracking of target VOCs (Dorokhov et al., 2014; Piqueret et al., 2023).

## MATERIALS AND METHODS

### Honeybee preparation

**Fig. 5.**
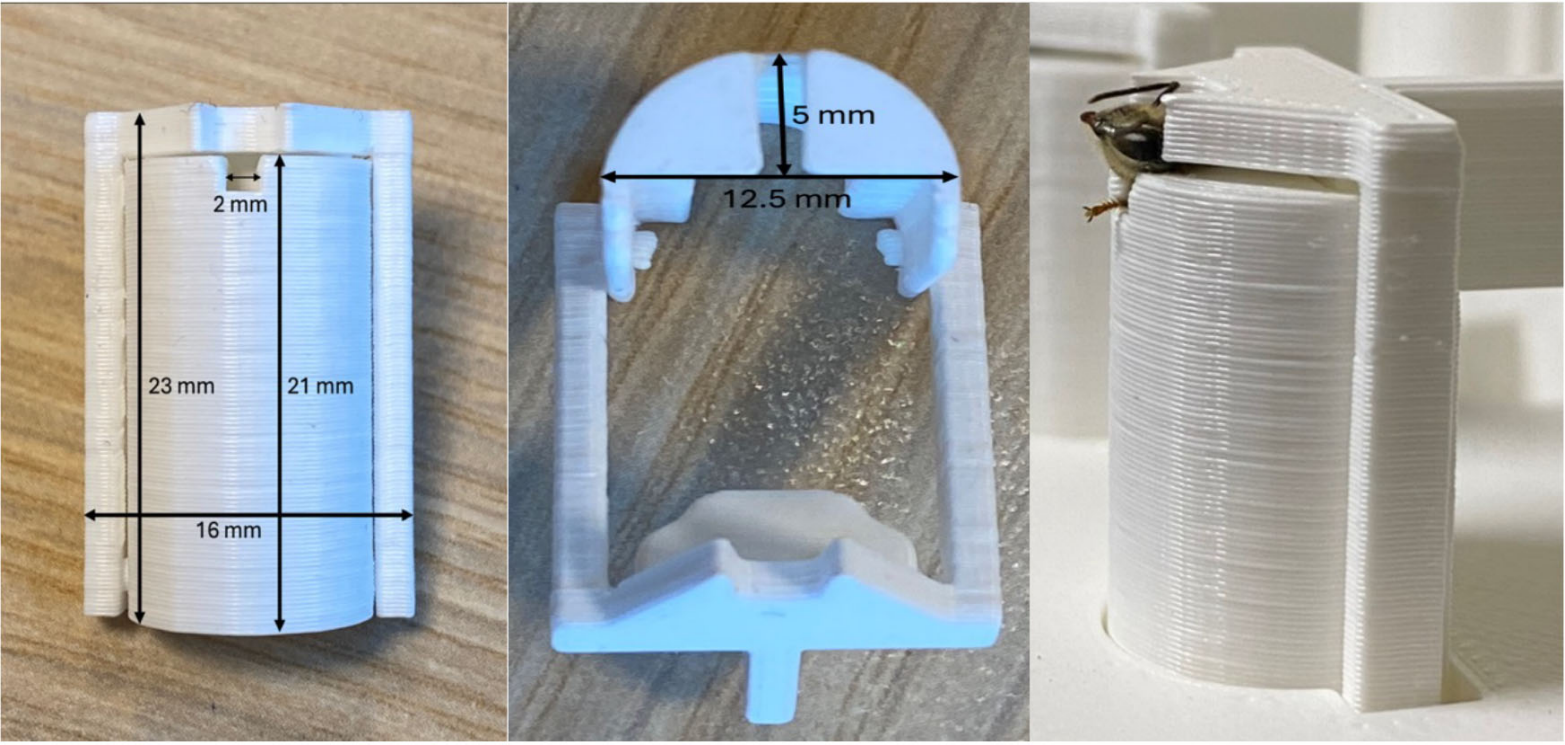
**Dimension of the bee harness, front (left picture) and back view (i.e. the lid; middle picture).** The picture on the right shows a harnessed bee.

We collected batches of forager honeybees (*Apis mellifera mellifera* or *Apis mellifera carnica*) from the same hive located in the Business and Science Park, Wageningen, The Netherlands, using a small vacuum device, between May and July 2023. The hive was treated with oxalic acid. The hive consisted of two boxes, where the younger bees and the queen were confined to the top box and the flight entrance was at the bottom. This design was meant to maximise the chance of selection of forager bees for the experiments. The bees were collected from the entrance of the hive as they departed or returned from foraging either in the morning (7:00-9:00 CET) or at the end of the afternoon (16:00-18:00 CET) depending on the protocol (see section below). The bees were likely a mixture of different ages. The bees were transported to the laboratory and sedated by placing them into a freezer (−18°C) for a maximum of 5 min (Kontos et al., 2022). Once sedated, the bees were carefully placed into a specifically designed harness inspired by Kontos et al. (2022) for testing (Fig. 5). The harness consisted of two parts, a half cylinder-shaped main chamber and a lid. The main chamber, where the bee body rested, was provided with a slight opening of 2 mm at the top where the bee neck was inserted. The outer frame, i.e. the lid, could be opened and closed to restrain the bee at the thorax level as well as behind the head. This secured the bee head while allowing its body to move freely. This new design carried improvements compared to Kontos et al. (2022), specifically concerning (i) the addition of the lid, which allowed to avoid the use of tape to keep the harness closed and gave more control to the user while harnessing a honeybee, and (ii) a wider main chamber (12.5 mm versus 10 mm) to allow the honeybee to move more freely without injuries.

After being harnessed, the bees rested at room temperature for 3 h (when collected in the morning of the behavioural assay) or overnight (when collected at the end of the afternoon of the day prior to the behavioural assay) before conditioning. When bees were collected for overnight starvation, they were fed *ad libitum* with a sugar solution (40%) before the night. When bees were collected in the morning, no feeding was done prior to the testing. To ensure the responsiveness of the bees, we touched their antennae with a cotton swab soaked in a 40% sugar solution and checked whether it elicited a PER response. Only bees that displayed a PER were selected for the conditioning and memory retention test. Each selected bee per day was uniquely identified with a number (ranging from 1 to 30). In total, 54 to 84 bees were tested per protocol (see Table 1).

### Olfactory conditioning and memory testing procedures

We tested three different bee training protocols (Table 3). In all protocols, the potato leaf samples (healthy and infected) were collected on the morning of the test to ensure freshness of the sample. The samples were collected from three healthy and three infected symptomatic PVY-infected potato plants, grown from healthy and infected tubers, respectively, all taken from one plant (cultivar Bintje, secondarily infected with PVY^NTN^ isolate 757), kept in a separate greenhouse compartment (insect-free, conditioned at 20±1°C, with additional LED lighting to extend day and night to 16 and 8 h, respectively). The intact leaflet samples were transported and kept in separate plastic bags to avoid any cross-contamination. Three leaves per plant were collected and alternated during conditioning to account for within-plant variability of volatile emission. The leaflet samples were then placed into a 20 ml plastic syringe (BD Discardit™ II, Becton Dickinson, Spain) and fixed at the height of the bee head using a fixed holder at a 2 cm distance between the bee and the syringe outlet, which released a 15 ml sample headspace volume. The syringe was manually operated to release an air puff containing the volatile compounds from potato leaflets (infected, healthy or empty) directly towards the bee head. The bees were rewarded with sugar (unconditioned stimulus, US) when exposed to infected samples in protocols A and B, and to healthy samples in protocol 1 (CS^+^, unconditioned stimulus given; CS^−^, unconditioned stimulus not given). The sample headspace was delivered in 5 s, with the sugar reward given 2 s after the start of the odour cue release for a total of 5 s (only in the case of CS^+^ cues). The sugary reward consisted of a 40% sucrose solution soaked on a cotton swab. The soaked swab was presented to the bee antennae first (a PER-triggering stimulus), and subsequently to the proboscis. Note that, in protocol A, the harnessed bee rested in the upward vertical for 25 s before the start of the delivery of the air puff and remained 22 s after the cue was delivered before switching to another individual. In protocols 1 and B, the harnessed bee rested in the upward vertical for 20 s before the start of the delivery of the air puff and remained 7 s after the cue was delivered before switching to another individual. The intertrial interval (ITI) [time elapsed between successive trials for the same bee (two trials per round)] was set at 10 min for all protocols. To keep track of the timing of the bee conditioning, we used a Tabata timer^®^.

**Table 3.** Details of the tested protocols with the starvation period, duration of the conditioning phase for one bee and one trial and the conditioned stimulus (CS^+^) and control (CS^−^)

Protocol	Starvation	Trial duration (per bee)	CS^+^ (sugar reward)	CS^−^ (no reward)
1	3 h (in the morning)	40 s	Healthy potato leaflet	Clean air (empty syringe)
A	Overnight	1 min	Infected potato leaflet	Healthy potato leaflet
B	3 h (in the morning)	40 s	Infected potato leaf disc	Healthy potato leaflet

The conditioning consisted of nine rounds. In each round, the focal bee was exposed to both healthy and infected potato leaf sample headspaces in a pseudorandomized order: C-T-C-T-T-C-T-C-C-T-T-C-C-T-C-T-T-C (control and treatment for empty and healthy sample, respectively, for protocol 1, and healthy and infected samples, respectively, for protocols A and B).

PER responses displayed by the bees were recorded during the first 3 s after odour delivery (before the sugar reward) regardless of the odour cue delivered and reward for analyses of their learning curves. The same was done during the memory retention test.

One hour after the last conditioning round, the memory retention test started. The same potato leaf samples used for the conditioning (hereafter infected_O, healthy_O) and freshly cut leaf samples, one healthy and one infected (hereafter healthy_F, infected_F), were used for testing memory retention. The bees were placed in an upward vertical position on the harness holder 2 cm away from the syringe outlet similarly as in the conditioning round. This time the tested sample headspaces were delivered without the sugar rewards in a pre-determined random manner. The PER responses of the bees were recorded to measure their capacity to distinguish the different odour cues previously learned.

### Analysis

We examined the learning curves of the bees for each protocol separately using a GLMM. These models use a binomial distribution and logit error link. The bees PER response (measured as either 0 or 1) served as the dependent variable (Zuur et al., 2009). The type of sample (CS^+^ and CS^−^), the conditioning rounds, and their interactions were considered explanatory variables (Kontos et al., 2022). If the interaction was not significant (*P*<0.05), it was removed from the model. We accounted for repeated measurements on individual bees by including their unique identification (bee ID) within day (date of testing) as a random intercept (Rabe-Hesketh and Skrondal, 2008). The models' assumptions were verified by residual analysis and qq-plots of the fixed and random effects (Hartig, 2019; Zuur et al., 2009). We conducted hypothesis testing using a chi-square distribution to evaluate the importance of the explanatory variables with a significance threshold set at *P*<0.05.

To assess the bee's discrimination accuracy between the tested samples after conditioning, we fitted again a GLMM with a binomial distribution and logit error link for each protocol (Zuur et al., 2009). In these models, the bees' response (PER: 0 or 1) was the dependent variable, while the type of sample (healthy_F, healthy_O, infected_F, infected_O, empty) was the fixed explanatory variable and the bee ID within days was introduced as a random intercept (Kontos et al., 2022). The models' assumptions were verified by residual analysis and qq-plots of the random intercepts (Hartig, 2019; Zuur et al., 2009). We conducted hypothesis testing using a chi-square distribution to evaluate the importance of the explanatory variables with a significance threshold set at *P*<0.05.

All data analyses were performed using the statistical software R version 4.3.1 (R Core Team, 2019). The GLMM were fit using the package lme4 (Bates et al., 2015), the Wald test was performed using the car package (Zeileis et al., 2008). The models' assumptions and validity were assessed with the package DHARMa (Hartig, 2019) for residual testing and with the package performance (Lüdecke et al., 2021) for the model fit calculations (i.e. r-squared). The post-hoc analysis was performed with emmeans (Russell, 2018) and the figures were drawn using the ggplot2 package (Wickham, 2016).

## Supplementary Material

10.1242/biolopen.061680_sup1 Supplementary information

## References

[BIO061680C1] Aartsma, Y., Bianchi, F. J. J. A., van der Werf, W., Poelman, E. H. and Dicke, M. (2017). Herbivore-induced plant volatiles and tritrophic interactions across spatial scales. *New Phytol.* 216, 1054-1063. 10.2307/90015824 28195346 PMC6079636

[BIO061680C2] Alves, L. H. S., Cassino, P. C. R. and Prezoto, F. (2015). Effects of abiotic factors on the foraging activity of Apis mellifera; Linnaeus, 1758 in inflorescences of *Vernonia polyanthes*; Less (Asteraceae). *Acta Sci.* 37, 405. 10.4025/actascianimsci.v37i4.27463

[BIO061680C3] Bates, D., Mächler, M., Bolker, B. M. and Walker, S. C. (2015). Fitting linear mixed-effects models using lme4. *J. Stat. Softw.* 67, 1-48. 10.18637/jss.v067.i01

[BIO061680C4] Bitterman, M. E., Menzel, R., Fietz, A. and Schäfer, S. (1983). Classical conditioning of proboscis extension in honeybees (*Apis mellifera*). *J. Comp. Psychol.* 97, 107-119. 10.1037/0735-7036.97.2.107 6872507

[BIO061680C5] Bridges, A. D., MaBouDi, H., Procenko, O., Lockwood, C., Mohammed, Y., Kowalewska, A., Romero González, J. E., Woodgate, J. L. and Chittka, L. (2023). Bumblebees acquire alternative puzzle-box solutions via social learning. *PLoS Biol.* 21, e3002019. 10.1371/journal.pbio.3002019 36881588 PMC9990933

[BIO061680C6] Bromenshenk, J. J., Henderson, C. B., Seccomb, R. A., Welch, P. M., Debnam, S. E. and Firth, D. R. (2015). Bees as biosensors: chemosensory ability, honey bee monitoring systems, and emergent sensor technologies derived from the pollinator syndrome. *Biosensors* 5, 678-711. 10.3390/BIOS5040678 26529030 PMC4697140

[BIO061680C7] Chamberlain, K., Briens, M., Jacobs, J. H., Clark, S. J. and Pickett, J. A. (2012). Use of honey bees (*Apis mellifera* L.) to detect the presence of Mediterranean fruit fly (*Ceratitis capitata* Wiedemann) larvae in Valencia oranges. *J. Sci. Food Agric.* 92, 2050-2054. 10.1002/JSFA.5742 22653619

[BIO061680C8] Chittka, L. and Rossi, N. (2022). Social cognition in insects. *Trends Cogn. Sci.* 26, 578-592. 10.1016/J.TICS.2022.04.001 35570086

[BIO061680C9] Decourtye, A., Devillers, J., Cluzeau, S., Charreton, M. and Pham-Delègue, M. H. (2004). Effects of imidacloprid and deltamethrin on associative learning in honeybees under semi-field and laboratory conditions. *Ecotoxicol. Environ. Saf.* 57, 410-419. 10.1016/J.ECOENV.2003.08.001 15041263

[BIO061680C10] Dorokhov, Y. L., Komarova, T. V. and Sheshukova, E. V. (2014). Volatile organic compounds and plant virus–host interaction. In *Plant Virus–Host Interaction* (ed. R. K. Gaur, Thomas Hohn, Pradeep Sharma), pp. 241-262. Elsevier. 10.1016/B978-0-12-411584-2.00013-5

[BIO061680C11] Dupuis, B., Nkuriyingoma, P. and Ballmer, T. (2024). Economic impact of *potato virus y* (PVY) in Europe. *Potato Res.* 67, 55-72. 10.1007/S11540-023-09623-X/TABLES/2

[BIO061680C12] Erber, J., Hoormann, J. and Scheiner, R. (2006). Phototactic behaviour correlates with gustatory responsiveness in honey bees (*Apis mellifera* L.). *Behav. Brain Res.* 174, 174-180. 10.1016/J.BBR.2006.07.023 16934881

[BIO061680C13] Filipi, J., Stojnić, V., Muštra, M., Gillanders, R. N., Jovanović, V., Gajić, S., Turnbull, G. A., Babić, Z., Kezić, N. and Risojević, V. (2022). Honeybee-based biohybrid system for landmine detection. *Sci. Total Environ.* 803, 150041. 10.1016/j.scitotenv.2021.150041 34500270

[BIO061680C14] Giurfa, M. (2007). Behavioral and neural analysis of associative learning in the honeybee: a taste from the magic well. *J. Comp. Physiol. A* 193, 801-824. 10.1007/s00359-007-0235-9 17639413

[BIO061680C15] Giurfa, M. and Sandoz, J. C. (2012). Invertebrate learning and memory: fifty years of olfactory conditioning of the proboscis extension response in honeybees. *Learn. Mem.* 19, 54-66. 10.1101/LM.024711.111 22251890

[BIO061680C16] Hartig, F. (2019). DHARMa: residual diagnostics for hierarchical (multi-level/mixed) regression models. *R Package Version 0.2, 4.* http://florianhartig.github.io/DHARMa/.

[BIO061680C17] Jiménez-Martínez, E. S., Bosque-Pérez, N. A., Berger, P. H., Zemetra, R. S., Ding, H. and Eigenbrode, S. D. (2004). Volatile cues influence the response of *Rhopalosiphum padi* (Homoptera: Aphididae) to barley yellow dwarf virus–Infected transgenic and untransformed wheat. *Environ. Entomol.* 33, 1207-1216. 10.1603/0046-225X-33.5.1207

[BIO061680C18] Karasev, A. V. and Gray, S. M. (2013). Continuous and emerging challenges of *potato virus y* in potato. *Annu. Rev. Phytopathol.* 51, 571-586. 10.1146/ANNUREV-PHYTO-082712-102332/CITE/REFWORKS 23915135

[BIO061680C19] Kontos, E., Samimi, A., der Honing, R. W. H.-V., Priem, J., Avargués-Weber, A., Haverkamp, A., Dicke, M., Gonzales, J. L. and Van Der Poel, W. H. M. (2022). Bees can be trained to identify SARS-CoV-2 infected samples. *Biol. Open* 11, bio059111. 10.1242/BIO.059111/275246 35502829 PMC9096705

[BIO061680C20] Laloi, D., Bailez, O., Blight, M. M., Roger, B., Pham-Delègue, M. H. and Wadhams, L. J. (2000). Recognition of complex odors by restrained and free-flying honeybees, *Apis mellifera*. *J. Chem. Ecol.* 26, 2307-2319. 10.1023/A:1005522826673/METRICS

[BIO061680C21] Laska, M., Galizia, C. G., Giurfa, M. and Menzel, R. (1999). Olfactory discrimination ability and odor structure–Activity relationships in honeybees. *Chem. Senses* 24, 429-438. 10.1093/CHEMSE/24.4.429 10480679

[BIO061680C22] Lüdecke, D., Ben-Shachar, M., Patil, I., Waggoner, P. and Makowski, D. (2021). performance: an R package for assessment, comparison and testing of statistical models. *J. Open Source Softw.* 6, 3139. 10.21105/joss.03139

[BIO061680C23] Menzel, R. and Bitterman, M. E. (1983). Learning by honeybees in an unnatural situation. In *Neuroethology and Behavioral Physiology* (ed F. Huber and H. Markl), pp. 206-215. Berlin Heidelberg: Springer. 10.1007/978-3-642-69271-0_15

[BIO061680C24] Ngumbi, E., Eigenbrode, S. D., Bosque-Pérez, N. A., Ding, H. and Rodriguez, A. (2007). *Myzus persicae* is arrested more by blends than by individual compounds elevated in headspace of plrv-infected potato. *J. Chem. Ecol.* 33, 1733-1747. 10.1007/S10886-007-9340-Z/TABLES/5 17680312

[BIO061680C25] Paoli, M. and Galizia, G. C. (2021). Olfactory coding in honeybees. *Cell Tissue Res.* 383, 35-58. 10.1007/S00441-020-03385-5 33443623 PMC7873095

[BIO061680C26] Petek, M., Rotter, A., Kogovšek, P., Baebler, Š., Mithöfer, A. and Gruden, K. (2014). *Potato virus Y* infection hinders potato defence response and renders plants more vulnerable to Colorado potato beetle attack. *Mol. Ecol.* 23, 5378-5391. 10.1111/mec.12932 25251011 PMC4237146

[BIO061680C27] Pham-Delègue, M. H., Blight, M. M., Kerguelen, V., Le Métayer, M., Marion-Poll, F., Sandoz, J. C. and Wadhams, L. J. (1997). Discrimination of oilseed rape volatiles by the honeybee: combined chemical and biological approaches. *Entomol. Exp. Appl.* 83, 87-92. 10.1046/J.1570-7458.1997.00160.X

[BIO061680C28] Piqueret, B., Sandoz, J.-C. and d'Ettorre, P. (2023). The neglected potential of invertebrates in detecting disease via olfaction. *Front. Ecol. Evol.* 10, 960757. 10.3389/fevo.2022.960757

[BIO061680C29] R Core Team. (2019). R: a language and environment for statistical computing. R Foundation for Statistical Computing, 1, 409.

[BIO061680C30] Rabe-Hesketh, S. and Skrondal, A. (2008). *Generalized Linear Mixed-effects Models*, 1st edn. Chapman and Hall/CRC.

[BIO061680C31] Russell, L. (2018). Emmeans: estimated marginal means, aka least-squares means. *R Package Version, 1(2)*.

[BIO061680C32] Sandoz, J. C., Pham-Delègue, M., Renou, M. and Wadhams, L. J. (2001). Asymmetrical generalisation between pheromonal and floral odours in appetitive olfactory conditioning of the honeybee (*Apis mellifera* L.). *J. Comp. Physiol. A* 187, 559-568. 10.1007/s003590100228 11730303

[BIO061680C33] Schneider, L., Rebetez, M. and Rasmann, S. (2022). The effect of climate change on invasive crop pests across biomes. *Curr. Opin. Insect Sci.* 50, 100895. 10.1016/j.cois.2022.100895 35240333

[BIO061680C34] Suckling, D. M. and Sagar, R. L. (2011). Honeybees *Apis mellifera* can detect the scent of *Mycobacterium tuberculosis*. *Tuberculosis* 91, 327-328. 10.1016/j.tube.2011.04.008 21546308

[BIO061680C35] Tholl, D., Boland, W., Hansel, A., Loreto, F., Röse, U. S. R. and Schnitzler, J. P. (2006). Practical approaches to plant volatile analysis. *Plant J.* 45, 540-560. 10.1111/J.1365-313X.2005.02612.X 16441348

[BIO061680C36] Tsedaley, B. (2015). A review paper on *potato virus y* (PVY) biology, economic importance and its managements. *J. Biol. Agric. Healthc.* 5, 110-126. www.iiste.org.

[BIO061680C37] Twidle, A. M., Mas, F., Harper, A. R., Horner, R. M., Welsh, T. J. and Suckling, D. M. (2015). Kiwifruit flower odor perception and recognition by honey bees, *Apis mellifera*. *J. Agric. Food Chem.* 63, 5597-5602. 10.1021/ACS.JAFC.5B01165/ASSET/IMAGES/MEDIUM/JF-2015-011657_0004.GIF 26027748

[BIO061680C38] Wickham, H. (2016). ggplot2: elegant graphics for data analysis. Springer-Verlag. 10.1007/978-3-319-24277-4

[BIO061680C39] Wright, G. A. and Smith, B. H. (2004). Different thresholds for detection and discrimination of odors in the honey bee (*Apis mellifera*). *Chem. Senses* 29, 127-135. 10.1093/CHEMSE/BJH016 14977809

[BIO061680C40] Zeileis, A., Kleiber, C. and Jackman, S. (2008). Regression models for count data in R. *J. Stat. Softw.* 27, 1-25. 10.18637/jss.v027.i08

[BIO061680C41] Zuur, A. F., Ieno, E. N., Walker, N. J., Saveliev, A. A. and Smith, G. M. (2009). *Mixed Effects Models and Extentions in Ecology with R*. Springer. 10.1007/978-0-387-87458-6

