# Supplementary material for: An ecological approach to honeybee olfactory conditioning: challenges and potential for the monitoring of potato virus Y infection
Source: Biol Open. 2025 Sep 15;14(9):bio061680. doi: 10.1242/bio.061680 (PMC12486206; doi:10.1242/bio.061680)
Supplement: Supplementary information [file biolopen-14-061680-s1.pdf]

Table S1. Results of the binomial generalized linear mixed-effects model (GLMM) for the conditioning phase of Protocol 1. The table reports effects with leaf sample type (clean, healthy F, and healthy O) with corresponding log-odds with standard errors (SE), Z-values (Z), and p-values (P). Random intercept information with the variance and standard deviation (SD) along with the model fit (Conditional R^2) are reported

**Predictor**	**Log-odds**	**SE**	**Z**	**P**
**Fixe part**				
Intercept [Clean air]	-2.63	0.23	-11.55	<0.001
Sample [Healthy]	1.13	0.15	7.45	<0.001
Conditioning round	0.07	0.03	2.46	0.014
**Random part**				
Variance	0.87	-	-	-
SD	0.93	-	-	-
Number of bee (total)	77	-	-	-
Number of observations	1386	-	-	-
Conditional R^2	0.27	-	-	-

Table S2. Results of the binomial generalized linear mixed-effects model (GLMM) for the conditioning phase of Protocol A. The table reports effects with leaf sample type (healthy F, and healthy O, Infected F, and Infected O) with corresponding log-odds with standard errors (SE), Z-values (Z), and p-values (P). Random intercept information with the variance and standard deviation (SD) along with the model fit (Conditional R^2) are reported

**Predictor**	**Log-odds**	**SE**	**Z**	**P**
**Fixe part**				
Intercept [Healthy]	-2.11	0.34	-6.14	<0.001
Sample [Infected]	1.13	0.17	6.56	<0.001
Conditioning round	0.05	0.03	1.54	0.120
**Random part**				
Variance	3.52	-	-	-
SD	1.88	-	-	-
Number of bee (total)	54	-	-	-
Number of observations	972	-	-	-
Conditional R^2	0.54	-	-	-

Biology Open • Supplementary information

Table S3. Results of the binomial generalized linear mixed-effects model (GLMM) for the conditioning phase of Protocol B. The table reports effects with leaf sample type (healthy F, and healthy O, Infected F, and Infected O) with corresponding log-odds with standard errors (SE), Z-values (Z), and p-values (P). Random intercept information with the variance and standard deviation (SD) along with the model fit (Conditional R^2) are reported.

**Predictor**	**Log-odds**	**SE**	**Z**	**P**
**Fixe part**				
Intercept [Healthy]	-3.10	0.29	-10.70	<0.001
Sample [Infected]	1.09	0.18	6.09	<0.001
Conditioning round	0.00	0.03	0.00	1.000
**Random part**				
Variance	1.88	-	-	-
SD	1.37	-	-	-
Number of bee (total)	84	-	-	-
Number of observations	1512	-	-	-
Conditional R^2	0.40	-	-	-

Table S4. Results of the binomial generalized linear mixed-effects model (GLMM) for the memory retention phase of Protocol 1. The table reports effects with leaf sample type (clean, healthy F, and healthy O) with corresponding log-odds with standard errors (SE), Z-values (Z), and p-values (P). Random intercept information with the variance and standard deviation (SD) along with the model fit (Conditional R^2) are reported.

**Predictor**	**Log-odds**	**SE**	**Z**	**P**
**Fixe Part**				
Intercept [Clean air]	-5.63	1.34	-4.18	<0.001
Sample [Healthy F]	4.44	1.24	3.57	<.0001
Sample [Healthy O]	4.44	1.24	3.57	<0.001
**Random Part**				
Variance	3.16	-	-	-
SD	1.78	-	-	-
Number of bee (total)	71	-	-	-
Number of observations	213	-	-	-
Conditional R^2	0.70	-	-	-

Table S5. Results of the binomial generalized linear mixed-effects model (GLMM) for the memory retention phase of Protocol A. The table reports effects with leaf sample type (healthy F, and healthy O, Infected F, and Infected O) with corresponding log-odds with standard errors (SE), Z-values (Z), and p-values (P). Random intercept information with the variance and standard deviation (SD) along with the model fit (Conditional R^2) are reported.

**Predictor**	**Log-odds**	**SE**	**Z**	**P**
**Fixe Part**				
Intercept [Healthy F]	-0.86	0.37	-2.33	0.020
Sample [Healthy O]	-0.22	0.46	-0.47	0.636
Sample [Infected F]	0.11	0.46	0.23	0.816
Sample [Infected O]	0.81	0.45	1.79	0.074
**Random Part**				
Variance	1.36	-	-	-
SD	1.17	-	-	-
Number of bee (total)	53	-	-	-
Number of observations	212	-	-	-
Conditional R^2	0.32	-	-	-

Table S6. Results of the binomial generalized linear mixed-effects model (GLMM) for the memory retention phase of Protocol B. The table reports effects with leaf sample type (healthy F, and healthy O, Infected F, and Infected O) with corresponding log-odds with standard errors (SE), Z-values (Z), and p-values (P). Random intercept information with the variance and standard deviation (SD) along with the model fit (Conditional R^2) are reported.

**Predictor**	**Log-odds**	**SE**	**Z**	**P**
**Fixed Part**				
Intercept [Healthy F]	-1.95	0.41	-4.74	<0.001
Sample [Healthy O]	0.46	0.43	1.06	0.287
Sample [Infected F]	-0.82	0.50	-1.65	0.098
Sample [Infected O]	0.19	0.44	0.44	0.661
**Random Part**				
Variance	2.38	-	-	-
SD	1.54	-	-	-
Number of bee (total)	83	-	-	-
Number of observations	332	-	-	-
Conditional R^2	0.44	-	-	-

Biology Open • Supplementary information
